# Western European Variation in the Organization of Esophageal Cancer Surgical Care

**DOI:** 10.1093/dote/doae033

**Published:** 2024-04-26

**Authors:** Maurits R Visser, Daan M Voeten, Suzanne S Gisbertz, Jelle P Ruurda, Michael P Achiam, Magnus Nilsson, Sheraz R Markar, Manuel Pera, Riccardo Rosati, Guillaume Piessen, Philippe Nafteux, Christian A Gutschow, Peter P Grimminger, Jari V Räsänen, John V Reynolds, Hans-Olaf Johannessen, Pedro Vieira, Michael Weitzendorfer, Aristotelis Kechagias, Mark I van Berge Henegouwen, Richard van Hillegersberg

**Affiliations:** Department of Surgery, University Medical Center Utrecht, Utrecht, the Netherlands; Scientific Bureau, Dutch Institute for Clinical Auditing, Leiden, the Netherlands; Department of Surgery, Amsterdam UMC location University of Amsterdam, Amsterdam, the Netherlands; Cancer Treatment and Quality of Life, Cancer Center Amsterdam, Amsterdam, the Netherlands; Department of Surgery, Amsterdam UMC location University of Amsterdam, Amsterdam, the Netherlands; Cancer Treatment and Quality of Life, Cancer Center Amsterdam, Amsterdam, the Netherlands; Department of Surgery, University Medical Center Utrecht, Utrecht, the Netherlands; Department of Surgery and Transplantation, Copenhagen University Hospital Rigshospitalet, Copenhagen, Denmark; Department of Upper Gastrointestinal Diseases, Karolinska University Hospital and Department of Clinical Science Technology and Interventions, Stockholm, Sweden; Nuffield Department of Surgical Sciences, University of Oxford, Oxford, UK; Section of Gastrointestinal Surgery, Hospital del Mar, Department of Surgery, Universitat Autònoma de Barcelona, Hospital del Mar Medical Research Institute (IMIM), Barcelona, Spain; Department of Gastrointestinal Surgery, IRCCS San Raffaele Scientific Institute, Milan, Italy; Department of Digestive and Oncological Surgery, Claude Huriez University Hospital, Lille, France; Department of Thoracic Surgery, University Hospitals Leuven, Leuven, Belgium; Department of Surgery and Transplantation, University Hospital Zurich, Zurich, Switzerland; Department of General, Visceral and Transplant Surgery, University Medical Center of the Johannes Gutenberg University Mainz, Mainz, Germany; Department of General Thoracic and Esophageal Surgery, Heart and Lung Centre, Helsinki University Hospital, Helsinki, Finland; Trinity St. James’s Cancer Institute, St. James’s Hospital, Dublin, Ireland; Department of Gastroenterological Surgery, Oslo University Hospital, Oslo, Norway; Digestive Cancer Unit, Champalimaud Clinical Centre – Champalimaud Foundation, Lisbon, Portugal; Department of Surgery, Paracelsus Medical University Salzburg, Salzburg, Austria; Department of Surgery, Athens Metropolitan Hospital, Athens, Greece; Department of Surgery, Amsterdam UMC location University of Amsterdam, Amsterdam, the Netherlands; Cancer Treatment and Quality of Life, Cancer Center Amsterdam, Amsterdam, the Netherlands; Department of Surgery, University Medical Center Utrecht, Utrecht, the Netherlands

**Keywords:** carcinoma, esophagus, surgery

## Abstract

Reasons for structural and outcome differences in esophageal cancer surgery in Western Europe remain unclear. This questionnaire study aimed to identify differences in the organization of esophageal cancer surgical care in Western Europe. A cross-sectional international questionnaire study was conducted among upper gastrointestinal (GI) surgeons from Western Europe. One surgeon per country was selected based on scientific output and active membership in the European Society for Diseases of the Esophagus or (inter)national upper GI committee. The questionnaire consisted of 51 structured questions on the structural organization of esophageal cancer surgery, surgical training, and clinical audit processes. Between October 2021 and October 2022, 16 surgeons from 16 European countries participated in this study. In 5 countries (31%), a volume threshold was present ranging from 10 to 26 annual esophagectomies, in 7 (44%) care was centralized in designated centers, and in 4 (25%) no centralizing regulations were present. The number of centers performing esophageal cancer surgery per country differed from 4 to 400, representing 0.5–4.9 centers per million inhabitants. In 4 countries (25%), esophageal cancer surgery was part of general surgical training and 8 (50%) reported the availability of upper GI surgery fellowships. A national audit for upper GI surgery was present in 8 (50%) countries. If available, all countries use the audit to monitor the quality of care. Substantial differences exist in the organization and centralization of esophageal cancer surgical care in Western Europe. The exchange of experience in the organizational aspects of care could further improve the results of esophageal cancer surgical care in Europe.

## INTRODUCTION

In 2011, the Dutch Upper Gastrointestinal Cancer Audit (DUCA) was established in the Netherlands, registering all patients undergoing surgery for esophageal or gastric cancer with the intent of resection.^1^ The goal of the audit was to monitor the quality of esophagogastric cancer surgery and improve outcomes through a reduction of variation between hospitals by providing surgical teams with benchmarked, hospital-specific process and outcome information.^1^^,^^2^ Throughout the first decade of its existence, the audit has had a positive impact on patient outcomes.^3^ Similarly, Denmark and the UK have reported a positive impact of their national audits.^4^^,^^5^

In addition to the impact on the quality of care, an audit provides invaluable information for research on national and international basis. Using audit and registry data, studies have shown significant variation in both treatment characteristics and surgical outcomes between Western European countries.^6–8^ A study comparing postoperative outcomes in the Netherlands, Ireland, and 24 high-volume European centers found significant differences in complication rates and 30-day mortality.^9^ In Ireland, 90-day mortality was reported as low as 0.9%, compared to the 4.5% and 2.4% in the European cohort and the Netherlands, respectively. Using data from the European Registration of Cancer Care initiative, Messager et al. also found large differences in 30-day and in-hospital mortality rates between the UK, the Netherlands, France, Spain, and Ireland.^10^

An important factor in these outcome differences is the structural organization of esophageal cancer surgical care. Next to clinical audits, other organizational characteristics, such as the centralization of care, have been shown to significantly impact results. Multiple studies reported on a positive volume–outcome relationship in esophageal cancer surgery, with lower mortality rates and superior survival rates after esophagectomy in high-volume centers.^6^^,^^11–14^ In some countries, these studies provoked a first wave of centralization with the introduction of minimum volume standards or centralization in designated centers. However, it is unknown to what extent centralization and other structural organizational characteristics have reached throughout Western Europe. Therefore, the aim of this study was to identify differences in the structural components and organization of esophageal cancer surgical care in Western Europe.

## METHODS

A cross-sectional international questionnaire study among upper gastrointestinal and esophageal surgeons from Western European countries was performed to evaluate the organization of esophageal cancer surgical care. One surgeon per country was approached to participate in the study. Surgeons were approached based on their scientific output in the field (guideline: >20 publications) as well as active membership in the European Society for Diseases of the Esophagus or (inter)national esophageal cancer care committee. These surgeons were asked to complete the questionnaire for their country or recommend a more suited colleague.

### Questionnaire

The questionnaire consisted of 51 structured questions describing the organization of the centralization of esophageal cancer surgery, surgical training, and clinical audits. The entire questionnaire is presented in Supplementary file S1. The following endpoints were selected for this study:

1. Differences in the organization of centralization of esophageal cancer surgery.
2. Differences in the availability and organization of clinical audits.
3. Differences in the organization of esophageal cancer surgical training for residents and fellows.

The primary outcome was the presence of surgical service centralization in Western Europe. Secondary outcomes evaluated included organizational characteristics of clinical audits, standards of clinical care, and surgical training.

### Definitions

A standard of clinical care was defined as a standard to which hospitals must comply to perform esophageal cancer surgery. Centralization of care consisted of three options:

1. Centralization in designated centers: the government is responsible for appointing the hospitals performing esophageal cancer surgery, not necessarily based on volume.
2. Minimum annual hospital volume threshold: hospitals are allowed to perform esophageal cancer surgery if they reach the annual volume threshold.
3. No national centralization: care is neither centralized in designated centers nor a volume threshold is present on an official national basis.

### Statistics

The questionnaire was conducted using Google Forms and figures made using Microsoft Excel. The results of the questionnaire were described qualitatively and using frequencies and percentages. No approval from a medical ethics committee was required for this study as no individual patient data were reported.

## RESULTS

The questionnaire was answered by 16 surgeons from 16 countries in Western Europe. One surgeon (1/16, 6%) suggested a colleague to participate in the study. All participating countries and their representing surgeon are shown in Supplementary table S2.

### Organization of esophageal cancer care

**Fig. 1:**
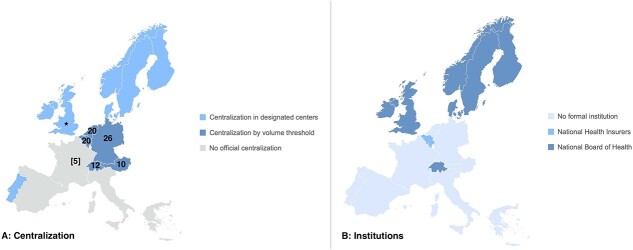
(A) The presence of centralization characteristics for esophageal cancer surgery in Western Europe. The numbers represent the national volume threshold. France is currently implementing a volume threshold of 5. (B) The institutions responsible for appointing hospitals to be able to perform esophageal cancer surgery. *In the UK, centers are divided to perform 50 esophagogastric resections per year. ^In the UK, the Association of Upper Gastrointestinal Surgeons is responsible.

Figure 1A shows the presence of an annual minimum hospital volume threshold or centralized care by the government in designated centers in Western Europe. In 12 (75%) countries, there is a form of centralization present, either through a minimum annual volume threshold (31%) or centralization in designated centers (44%). In the UK, centers are divided based on the served population to perform ~50 combined esophagogastric resections per year. France had a volume threshold of 30 digestive oncological surgeries, regardless of the organ, and is currently implementing an annual volume threshold of 5 for esophageal cancer surgery. The institution responsible for the enforcement of centralization was reported as either the government, health care insurers, or the national association of surgeons. In Austria and Switzerland, the minimum volume threshold is not actively enforced, resulting in centers performing esophageal cancer surgery but not meeting the volume threshold. Portugal has officially centralized esophageal cancer surgery to 6 hospitals in 2017, but since this centralization is not mandatory, 4 more centers perform esophageal surgery. No countries reported volume thresholds based on surgeon volume.

Table 1 shows the centralization characteristics and volume thresholds per country. Current volume thresholds range from 10 to 26. The range of centers performing esophagectomies is 4–400, with France and Germany reporting ~308 and 400 centers, respectively. All countries had between 0.5 and 4.9 centers per million inhabitants.

**Table 1:** Centralization characteristics per country

	**Centralization**	**National volume threshold**	**Number of centers** ^†^	**Centers per million inhabitants** ^‡^
Austria	Volume threshold	10	12^‡‡^	1.3
Belgium	Volume threshold	20	10	0.8
Germany	Volume threshold	26^§^	400	4.9
Switzerland	Volume threshold	12	8^‡‡^	1
The Netherlands	Volume threshold	20	15	0.9
Denmark	Designated centers	(35–130)	4	0.7
Finland	Designated centers	(5–50)	6	1.0
Ireland	Designated centers	(20–80)	4	0.8
Norway	Designated centers	(20–60)	4	1.4
Portugal	Designated centers	(10–50)	6^‡‡^	0.6
Sweden	Designated centers	(15–60)	7	0.7
UK	Designated centers	(50)^¶^	32	0.5
France	Imminent volume threshold	[5]^††^	308	4.5
Greece	None	—	10	0.9
Italy	None	—	136	2.2
Spain	None, regional differences	—	80	1.6

^†^Number of centers performing esophageal cancer surgery

^‡^Number of inhabitants in 2020

§ Germany raised the volume threshold to 26 in 2022

¶ Centralized in designated centers on population size to perform 50 esophagogastric resections

^††^France is implementing a volume threshold of 5

^‡‡^Official number of centers. However, additional centers perform esophageal cancer surgery despite them not meeting the centralization criteria

In 8 (50%) of the 16 countries, hospitals are allowed to decide for themselves whether to perform esophageal cancer surgery (Fig. 1B). In the remaining 8 countries (50%), hospitals are appointed by either the National Board of Health or National Health Insurers.

Most countries reported future perspectives toward further centralization of esophageal cancer surgery. Germany realized a raise from 13 to 26 operations per year in 2022. In the Netherlands, a national health care agreement was released in 2022, containing the ambition for introducing minimum volume thresholds of 50 or 100 operations per year.^15^ Esophageal cancer surgery is centralized by the government in Belgium, Denmark, Finland, Ireland, Norway, Switzerland, Sweden, and the UK, with all countries reporting the evaluation of results in the upcoming years. Although 3 regions are centralized in Spain, with one having a volume threshold of 11 operation per year, no future perspectives are reported for centralization in the remaining 14 regions. Mainly due to geographical factors, there are no plans for further centralization in Norway. Italy has no official national or regional plans for centralization, although surgeons in high-volume centers are pushing toward it. No future perspectives for further centralization were reported in Austria, Greece, and Portugal.

In 6 countries (France, Germany, the Netherlands, Portugal, the UK, and Sweden; 38%), patients with T4 tumors are referred to dedicated tertiary esophagectomy expert centers. Both colonic interpositions and patients with certain postoperative complications (i.e. tracheal fistula) are referred in 10 countries (63%). Four countries (Belgium, Denmark, Norway, and Switzerland; 25%) reported no specific referral system, as esophageal cancer surgery is already centralized in a limited number of hospitals. Denmark reported referral for advanced endoscopic resections. Only Austria and Italy reported no official referral criteria in non-centralized care.

**Fig. 2:**
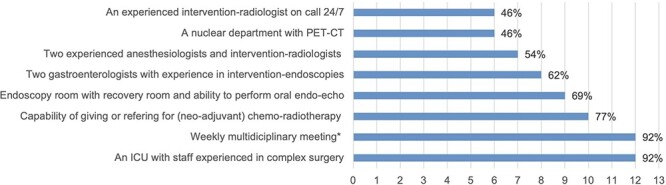
Requirements in clinical care standards for esophageal cancer surgery in 13 countries with clinical standards: Austria, Belgium, Denmark, Finland, France, Greece, Ireland, Norway, Portugal, Sweden, Switzerland, the Netherlands, and the UK. Multiple choice options were derived from the Dutch SONCOS standard^^^, with the possibility of adding additional requirements. *Consisting of at least one each: surgeons, gastroenterologists, oncologists, radiologists/nuclear specialists, oncological radiotherapists, pathologists, and (oncological) nurses. ^Stichting Oncologische Samenwerking (SONCOS), 2021, Multidisciplinaire normering oncologische zorg in Nederland

In only 3 countries (19%), namely, Germany, Spain, and Italy, no official standard of clinical care is present for centers performing esophageal cancer surgery. All other countries (81%) reported a national standard, with details shown in Figure 2.

The availability of a specialized upper gastrointestinal surgeon on call 24/7 in all esophagectomy centers was reported in 6 countries (38%; Austria, Belgium, Denmark, Ireland, Greece, and Switzerland), in most centers in 7 countries (50%), and in only a minority of centers in 3 (21%; Italy, the Netherlands, and Portugal).

### Training of surgeons

**Fig. 3:**
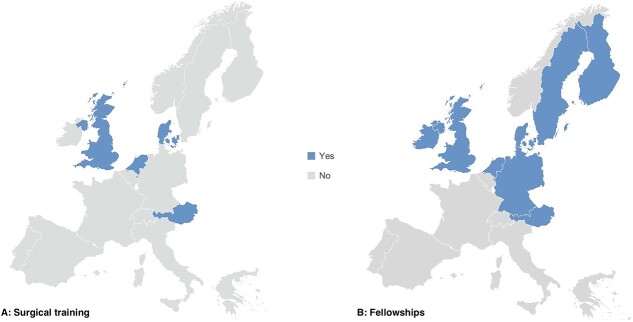
(A) Countries in Western Europe where esophageal cancer surgery is part of the final years of surgical training/residency. (B) Countries in Western Europe where organized upper gastrointestinal cancer surgery fellowships are available

Figure 3A shows in which 4 countries (25%) esophageal cancer surgery is part of (the final years of) general surgical training. Upper gastrointestinal (GI) surgeon certification is available in 31% of countries, with Denmark, Finland, the Netherlands, and Sweden using the European Union of Medical Specialties^16^ criteria. The other countries reported no specific criteria to be regarded as an upper gastrointestinal surgeon. Half of the countries reported the availability of organized upper gastrointestinal surgery fellowships in their country (Fig. 3B).

### Clinical audit

**Fig. 4:**
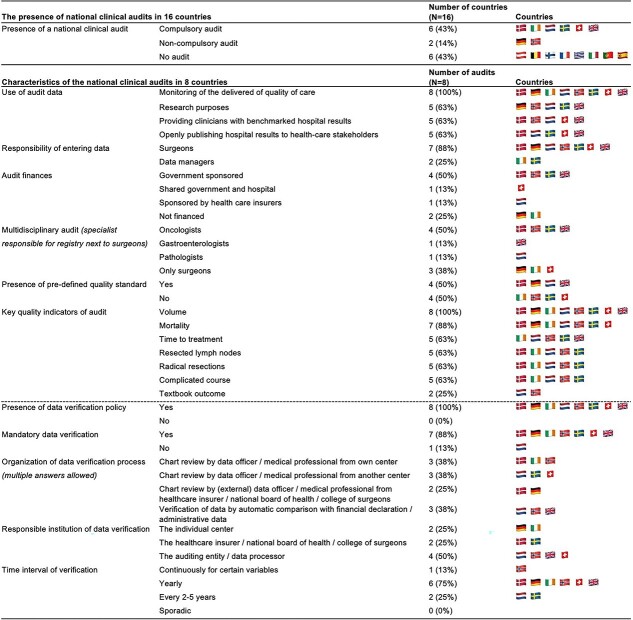
Characteristics of surgical clinical audits

Figure 4 shows the characteristics of national clinical audits focusing on upper gastrointestinal surgery per country in 8 countries (50%) in Western Europe. All audits registered patients and outcomes on hospital level. The UK also reports on surgeon details. All audits are used for monitoring of the delivered quality of care. Denmark, Germany, the Netherlands, and the UK (50%) reported the presence of clear-cut and predefined quality standards. In case these quality standards are not reached, hospitals either receive feedback or are visited by the audit committee. Key quality indicators were reported to be present in all countries with audits. The most common indicators were hospital volume (100%) and mortality (88%), with Textbook Outcome^17^ (25%) as the least used quality indicator.

In all audits, a data verification policy is established (Fig. 4). The structure of this process and the responsible institution differs per audit. Most audits (75%) perform the data verification process yearly.

## DISCUSSION

This study investigated the organization of esophageal cancer surgical care in 16 countries in Western Europe. We found a large variation in the organization of care, most notably in centralization through the implementation of volume thresholds or centralization in designated centers, and in the presence of a surgical clinical audit. In general, countries in North-Western Europe and Scandinavia more often implemented centralization and auditing than those in South-Western Europe.

One important aspect of the organization of surgical care is centralization, which directly results in an increase in-hospital volume. The two main strategies for centralization are dedicated centers appointed by the government, offering the strictest form of centralization, or minimum annual volume thresholds. Centers that (consistently) fail to meet the minimum volume threshold are reprimanded by the responsible institutions (healthcare insurers, national board of health or surgical association) to increase the annual volume or are forced to seize performing esophageal cancer surgery. These measures decreased the number of centers in centralized countries. In the Netherlands, the number of centers subsequently dropped from 24 in 2012 to 15 in 2022. Most countries with a centralization strategy have around 1 center per 1.4 million inhabitants or less, compared to a higher number of centers per million inhabitants in countries without centralization. Exceptions such as Greece are present, where there is a tendency to refer patients to 4 major centers, resulting in a limited number of centers without an official centralization strategy.

As one of the frontrunners in Europe, Denmark centralized esophageal cancer care in a few dedicated centers in 2003. An evaluation in 2017 showed a dramatic increase in lymph node yield and significant decreases in anastomotic leakage and mortality rates.^4^ Hospital volume has widely been accepted as an important determinant of complications, hospital mortality, and survival in complex gastrointestinal surgery.^6^^,^^11^^,^^12^^,^^14^^,^^18–20^

However, the extent of centralization does remain a matter of debate. A German study reported a volume-mortality relationship where the expected mortality was below the national average after 26 complex esophageal surgeries per year, resulting in the increase of the volume threshold from 13 to 26 in Germany.^21^ In the Netherlands, a positive volume–outcome relationship was seen in multiple outcome measures, including mortality and anastomotic leakage rates.^12^^,^^14^^,^^19^ Textbook outcome and 6-month and 2-year survival increased at higher volumes, plateauing ~40–60 annual esophagectomies. In the most recent study, lymph node yield and anastomotic leakage rate improved beyond 60 annual esophagectomies.^19^ These volume thresholds could be optimal for postoperative outcomes; however, the possible implementation is subject to many organizational and structural challenges.^22^

In this study, most countries reported future perspectives toward further centralization. Countries could benefit from previous experience in the introduction of centralization. Also, previous experience in mandating centralization could serve countries like Austria, Switzerland, and Portugal, reporting additional centers performing esophageal cancer surgery, despite them not meeting the centralization criteria.

Not only the increase in surgical experience from performing more procedures matters, but also ‘hospital resources’, like the 24/7 availability of intervention radiologists, surgeons, intensive care unit staff, and anesthesiologists experienced in complex gastrointestinal surgery, are important factors.^20^^,^^23^ Patients undergoing complex surgery need complex perioperative care, early recognition, and complex management of life-threatening complications. Higher mortality rates in low-volume hospitals are suggested to be caused by failure to rescue, and not only because of a higher incidence of complications.^24^ These hospital resources are included in the Eueopean CanCer Organization (ECCO) essential requirements for quality cancer care and standards for clinical care for centers performing esophageal cancer surgery.^25^ In this study, 81% of the countries reported the existence of an official standard and clear overlap was seen in the requirements between countries. As a clinical care standard usually accompanies centralization, these standards may play a role in the volume–outcome relationship by ensuring that all hospitals have adequate resources to perform complex surgery.

Multiple studies have shown the benefits of clinical audits, as they offer insights into the delivered quality of care, while also offering an invaluable tool for population-based clinical research.^3^^,^^4^^,^^26^ As the introduction of national audits was usually accompanied by centralization, the reported improvement of surgical outcomes in these countries can be partly attributed to these factors, as well as improvements in Early Rehabilitation After Surgery protocols, minimally invasive surgery, surgical advancements, and hospital recourses. Only 50% of Western European countries have a national clinical audit, indicating that these are not evenly implemented within Europe. Implementing a national clinical audit is challenging, and registration is time consuming, possibly limiting the distribution across Europe. In an audit, the integrity of data is paramount, requiring thorough validation.^27^ Every audit has a process for data verification implemented. However, the structure of these data verification procedures can differ significantly across audits.

In this study, all audits control hospital volume and most provide key quality indicators, like mortality and complications. However, only 25% of audits use the composite outcome measure Textbook Outcome. Textbook Outcome has been established as an important predictor for long-term survival.^28^ Key quality indicators like time-to-treatment, Textbook Outcome, severe complications, and other factors of the clinical care pathway, like MDT board review, are arguably as effective in measuring quality of care as hospital volume and (90/30-day/in-hospital) mortality. More audits should strive to implement these quality indicators to increase comparability.

National audits using uniform complication definitions, like those developed by the Esophageal Complications Consensus Group, also provide tools for comparing outcomes between countries.^7^ A DUCA study showed that the complication and anastomotic leakage rates were significantly higher in the Netherlands than in a multicenter international cohort.^7^^,^^8^ This led to the introduction of the first best-practice meetings for safe sharing of treatment outcomes in the Netherlands to induce nationwide improvement.^19^ In conjunction with centralization, these meetings are suggested to contribute to the decreasing anastomotic leakage rates in the Netherlands. During the 8 years since inception, pathological outcomes improved and mortality decreased after esophagectomy, showing the effectiveness of clinical auditing and the DUCA as an important tool for quality improvement in the Netherlands.^3^

Training of young surgeons could also be considered an important part of the organization of surgical care. In recent years, the European Society for Diseases of the Esophagus and Upper GI International Robotic Association have stressed the need for more accessible surgical training programs on upper gastrointestinal surgery. This study shows that these training programs are moderately available across Europe. In only 25% of countries, esophageal cancer surgery is part of general surgical training and in 50% of countries fellowships are available, mostly in North-Western Europe. Fellowships are invaluable to be trained in complex surgery on a high-volume basis and are believed to positively impact surgical outcomes, such as lower conversion rates in surgeons with minimally invasive fellowship training than those without.^29^ Although the attending fellows are sometimes multinational, the availability of training programs and fellowships for esophageal cancer surgery is a key area for improvement across Europe.

This study has some limitations. As the questionnaire was completed by only one surgeon per country, there was no control mechanism for the accuracy of the data. However, all participating surgeons are experienced surgeons with leading positions in their national organizations and interpretability of the questions was reduced as much as possible.

This study is the first study providing an overview of the organization of esophageal cancer surgical care in Europe. It could help identify areas for countries seeking to improve their esophageal cancer surgical care quality. Although differences in healthcare systems exist throughout Europe, these organizational characteristics could be implemented in most countries. Further studies could be conducted to assess the impact of these organizational characteristics by matching these results to surgical outcomes and mortality in Western Europe.

In conclusion, this study shows substantial differences in the organization of esophageal cancer surgical care between countries in Western Europe. This may reflect into the differences observed in the outcome of esophageal cancer care between countries. More European countries should strive toward the implementation of centralization and clinical audits, capitalizing on previous experience in centralizing esophageal cancer surgical care. The European societies could play an important role by stimulating this exchange of experience between countries. Countries with further advancements on centralization and clinical audits could serve as an example in developing organizational aspects of care and the implementation of national audits to further improve the results of esophageal cancer surgical care in Europe.

## Supplementary Material

Supplementaries_doae033

## Notes

Specific author contributions: Maurits Visser (Conceptualization, Data curation, Formal analysis, Investigation, Methodology, Visualization, Writing—original draft), Daan Voeten (Conceptualization, Methodology, Visualization, Writing—original draft, Writing—review & editing), Suzanne Gisbertz (Conceptualization, Formal analysis, Methodology, Supervision, Writing—review & editing), Jelle Ruurda (Conceptualization, Formal analysis, Methodology, Supervision, Writing—review & editing), Michael Achiam (Data curation, Investigation, Validation, Writing—review & editing), Hans-Olaf Johannessen (Data curation, Investigation, Validation, Writing—review & editing), Pedro Vieira (Data curation, Investigation, Validation, Writing—review & editing), Michael Weitzendorfer (Data curation, Investigation, Validation, Writing—review & editing), Aristotelis Kechagias (Data curation, Investigation, Validation, Writing—review & editing), Magnus Nilsson (Data curation, Investigation, Validation, Writing—review & editing), Sheraz R. Markar (Data curation, Investigation, Validation, Writing—review & editing), Manuel Pera (Data curation, Investigation, Validation, Writing—review & editing), Riccardo Rosati (Data curation, Investigation, Validation, Writing—review & editing), Christian Gutschow (Data curation, Investigation, Validation, Writing—review & editing), Guillaume Piessen (Data curation, Investigation, Validation, Writing—review & editing), Philippe Nafteux (Data curation, Investigation, Validation, Writing—review & editing), Peter Grimminger (Data curation, Investigation, Validation, Writing—review & editing), Jari Räsänen (Data curation, Investigation, Validation, Writing—review & editing), John Reynolds (Data curation, Investigation, Validation, Writing—review & editing), Mark van Berge Henegouwen (Conceptualization, Data curation, Formal analysis, Investigation, Methodology, Supervision, Writing—review & editing), and Richard van Hillegersberg (Conceptualization, Data curation, Formal analysis, Investigation, Methodology, Supervision, Validation, Writing—review & editing)
