# Supplementary material for: Western European Variation in the Organization of Esophageal Cancer Surgical Care
Source: Dis Esophagus. 2024 Apr 26;37(9):doae033. doi: 10.1093/dote/doae033 (PMC11360861; doi:10.1093/dote/doae033)
Supplement: Supplementaries_doae033 [file supplementaries_doae033.docx]

**Supplementary file 1: questionnaire.**

1. **Part I: Volume thresholds**
   1. Is there presently a minimum annual hospital volume-threshold for esophageal cancer surgery? *Yes / No*
      1. If yes, how high is the annual minimum hospital volume threshold? (please provide an exact number)
      2. If so; how is the threshold audited / enforced?
         - *The volume threshold is not actively enforced/audited*
         - *By the government*
         - *By the association of (upper GI) surgeons*
         - *By health-care insurers*
         - *Other…*

- 1. Is there presently a minimum annual surgeon volume-threshold for esophageal cancer surgery?  *Yes / No*
     1. If yes, how high is the annual minimum surgeon volume threshold? (please provide an exact number)
     2. If so; how is the threshold audited / enforced?
        - *The volume threshold is not actively enforced/audited*
        - *By the government*
        - *By the association of (upper GI) surgeons*
        - *By health-care insurers*
        - *Other…*
  2. Is there a minimum number of specialized upper GI surgeons per esophagectomy center? If yes, how many? *Yes / No*

- 1. How many esophagectomy resection centers are presently performing esophageal cancer surgery in your country?
     1. When is an esophageal cancer surgical center considered to be high volume in your country? (please provide an exact number)
     2. What is, approximately, the range of the annual esophageal cancer surgery volume in these centers? (please provide a range of exact numbers, i.e. 20 to 120)
  2. Are there future perspectives towards further centralization of esophageal cancer surgery? *Yes / No*
     1. If so, please explain. When is centralization expected? Towards how many centers? What is the expected volumes of these centers? What where the deciding factors towards centralization? Which authority is organizing and controlling the process?
  3. Are there different hospital types? (Multiple answers are possible)
- *Yes, hospitals are subdivided in esophagectomy expert and non-expert centers*
- *Yes, hospitals are subdivided into university and non-university hospitals*
- *Yes, hospitals are subdivided into private and public hospitals*
- *Yes, hospitals are subdivided into general hospitals and oncologic hospitals*
- *Other*
  1. If dedicated esophagectomy expert centers are available,
     1. What types of patients are referred to such centers?
     - *T4 Tumors*
     - *Colonic interpositions*
     - *Certain postoperative complications (i.e. tracheal fistula)*
     - *Other…*
     1. What percentage of hospitals is such a center? (please provide a percentage)
     2. What percentage of patients is treated in such a center? (please provide a percentage)
  2. Does each esophageal cancer center have a pre-specified geographical catchment/market area? *Yes / No*
     1. If yes, how are these catchment areas defined? i.e. based on:
        - *Number of inhabitants*
        - *State borders*
        - *Square meters*
        - *Number of esophageal cancer patients*
        - *Other…*
  3. Is a specialized upper gastrointestinal surgeon available or on call 24/7 in esophagectomy centers?
     - *Yes, in every esophagectomy center*
     - *Yes, in most centers but not in all*
     - *No, 24/7 surgical upper GI expertise is available only in a minority of centers*
     - *No, 24/7 surgical upper GI expertise is available in no centers at all*
     - *Other…*
  4. Can any hospital in your country decide for themselves whether they perform esophageal cancer surgery? *Yes / No*
     1. If not; who / which institution is responsible for appointing hospitals that can perform esophageal cancer surgery?

(If esophageal cancer surgery is not centralized to specialized centers, please answer: every center can perform esophageal cancer surgery)

- 1. Is there a standard for centers performing esophageal cancer surgery (like the SONCOS-standard)? If so, does it state some of the following requirements for these centers? (multiple answers are possible) *Yes / No*
     - A fully equipped endoscopy room with recovery room and the ability to perform oral endo-echo
     - At least two gastroenterologists with experience in intervention-endoscopies
     - At least two anesthesiologists and two intervention-radiologists with proven expertise on big gastrointestinal or oncological surgery
     - An experienced intervention-radiologist on call 24/7
     - An ICU with staff experienced in care after big gastrointestinal or oncological surgery
     - A Nuclear department with PET-CT
     - A center has to be able to give (neo-adjuvant) chemo-radiotherapy or refer patients to a center that has that ability
     - There is a weekly multidisciplinary meeting consisting of at least one each: surgeons, gastroenterologists, oncologists, radiologists/nuclear specialists, oncological radiotherapists, pathologists, case managers and (oncological) nurses
     - Other…
  2. How are new techniques implemented? E.g. when implementing minimally invasive surgery: is there a minimum volume threshold of minimally invasive resections? Is it controlled by a medical ethics committee?

1. **Part II: Organization of esophageal cancer surgical training**
   1. Is esophageal cancer surgery part of the (final years) of general surgical training / residency? *Yes / No*
      1. How long is this differentiation? (i.e. how many upper GI surgical procedures need to be performed? How many years?)
   2. Is upper GI surgeon certification available in your country? *Yes / No*
      1. If yes, are UEMS criteria used? *Yes / No*
      2. Are there national exams? Yes / No
   3. When can you become a certified upper GI surgeon?
      1. *During general surgical training / residency*
      2. *You can only become an upper GI surgeon after having completed general surgical training*
      3. *After performing … surgeries during the differentiation period of general surgical training*
      4. *Other…*
   4. Are organized upper GI fellowships available in your country? *Yes / No*
2. **Part III: National audit**
   1. Is there a national clinical audit focusing on upper gastrointestinal surgery?
      1. *Yes there is a mandatory surgical audit*
         - Which institution mandates that every patient is registered in an audit?
      2. *Yes there is an audit but it is not mandatory*
      3. *No*
   2. Are patients and outcomes of care registered at surgeon or hospital level?
      1. *Surgeon*
      2. *Hospital*
      3. *Other…*
   3. What is the audit data used for? (Multiple answers are possible)
      1. *Research purposes*
      2. *Monitoring of the delivered of quality of care*
         - Who is responsible for the monitoring of results?
      3. *Providing clinicians with benchmarked hospital results (i.e. availability of dashboards / reports that shows clinician their individual results compared to the national results or a predefined benchmark)*
      4. *Openly/transparently publishing hospital results to healthcare stakeholders (like insurers, patient federations etc.)*
      5. *Other…*
   4. Who is responsible for entering data into the audit database?
      1. *Surgeons themselves*
      2. *Specialized registrars*
      3. *Other…*
   5. How is the audit organized with regards to finances?
      1. Is the audit government-sponsored? *Yes / No*
      2. Do the individual hospitals / surgeons receive payment for entering data in the audit database? *Yes / No*
   6. Is the audit multidisciplinary?
      1. *Yes, it includes radiotherapists*
      2. *Yes, it includes gastroenterologists*
      3. *Yes, it includes oncologists*
      4. *Yes, it includes…*
      5. *No*
   7. Is the data actually audited? *Yes / No*
      1. If yes, which institution is responsible for the audit?
      2. Are there clear-cut and predefined quality standards? *Yes / No*
      3. What happens if one does not reach on of these quality standards (e.g. what happens when for example the postoperative mortality percentage is too high)
         1. Visits from audit committee
         2. Mandatory training
         3. Sanctions
         4. Other…
   8. What are the key quality indicators used in the audit in your country?
      1. Volume
      2. Time to treatment
      3. The amount of resected lymph nodes per patient
      4. Percentage of radical resections
      5. Percentage of patients with a complicated course
      6. Percentage of patients with textbook outcome
      7. Mortality
      8. Other…
3. **Part IV: Quality improvement**
   1. In some countries there are programs for increasing the quality of care. Is there a program for national quality improvement in your country? *Yes / No*
      1. If yes, please describe this program

| **Surgeon** | **Country** | **(Inter)national board memberships** |
| --- | --- | --- |
| Dr. Michael Weitzendorfer | Austria | Austrian Society of Surgical Oncology |
| Prof. Philippe Nafteux | Belgium | ESDE^A^, Royal Belgian Society for Surgery, Belgian Oncology Society |
| Dr. Michael P. Achiam | Denmark | Danish Esophagus Gastric Cancer Group, Upper GI section Danish Surgical Society |
| Dr. Jari Räsänen | Finland | UEMS^B^ secretary of board Thoracic Section |
| Prof. Guillaume Piessen | France | FRench EsoGastric Tumor working Group (FREGAT) |
| Prof. Peter P. Grimminger | Germany | ESDE^A^, ISDE^C^, CAOGI^D^, Upper GI International Robotic Association |
| Dr. Aristotelis Kechagias | Greece | - |
| Prof. John V. Reynolds | Ireland | ESDE^A^, ISDE^C^ |
| Prof. Riccardo Rosati | Italy | Former ESDE^A^, San Raffaele Comprehensive Cancer Center |
| Dr. Hans-Olaf Johannessen | Norway | - |
| Dr. Pedro Vieira | Portugal | - |
| Prof. Manuel Pera | Spain | Former ESDE^A^ and ISDE^C^, EURECCA^E^ |
| Prof. Magnus Nilsson | Sweden | ESDE^A^, ISDE^C^, Swedish National Registry of Esophageal and Gastric Cancer |
| Prof. Christian A. Gutschow | Switzerland | European Foregut Society |
| Prof. Mark I. van Berge Henegouwen | the Netherlands | Dutch Upper Gastrointestinal Cancer Audit, Upper GI Surgical Working Group Netherlands |
| Prof. Sheraz R. Markar | United Kingdom | ESDE^A^, ISDE^C^, EAES^F^, Association of Upper Gastrointestinal surgeons for Great Britain and Ireland |
| A. European Society for Diseases of the Esophagus. B. European Union of Medical Specialties (UEMS) C. International Society for Diseases of the Esophagus. D. Surgical workgroup upper gastrointestinal tract Germany. E. European Registration of Cancer Care. F. European Association of Endoscopic Surgery | | |

Table S2: Overview of representing surgeon per country in Western Europe.
